# A HIV-Tat/C4-binding protein chimera encoded by a DNA vaccine is highly immunogenic and contains acute EcoHIV infection in mice

**DOI:** 10.1038/srep29131

**Published:** 2016-06-30

**Authors:** Khamis Tomusange, Danushka Wijesundara, Jason Gummow, Tamsin Garrod, Yanrui Li, Lachlan Gray, Melissa Churchill, Branka Grubor-Bauk, Eric J. Gowans

**Affiliations:** 1 Virology Laboratory, Basil Hetzel Institute, Discipline of Surgery, University of Adelaide, Adelaide, South Australia, Australia; 2 Royal Australasian College of Surgeons, Adelaide, South Australia, Australia; 3 Centre for Biomedical Research, Burnet Institute, Melbourne VIC, Australia; 4 Department of Infectious Diseases, Monash University, Melbourne VIC, Australia

## Abstract

DNA vaccines are cost-effective to manufacture on a global scale and Tat-based DNA vaccines have yielded protective outcomes in preclinical and clinical models of human immunodeficiency virus (HIV), highlighting the potential of such vaccines. However, Tat-based DNA vaccines have been poorly immunogenic, and despite the administration of multiple doses and/or the addition of adjuvants, these vaccines are not in general use. In this study, we improved Tat immunogenicity by fusing it with the oligomerisation domain of a chimeric C4-binding protein (C4b-p), termed IMX313, resulting in Tat heptamerisation and linked Tat to the leader sequence of tissue plasminogen activator (TPA) to ensure that the bulk of heptamerised Tat is secreted. Mice vaccinated with secreted Tat fused to IMX313 (pVAX-sTat-IMX313) developed higher titres of Tat-specific serum IgG, mucosal sIgA and cell-mediated immune (CMI) responses, and showed superior control of EcoHIV infection, a surrogate murine HIV challenge model, compared with animals vaccinated with other test vaccines. Given the crucial contribution of Tat to HIV-1 pathogenesis and the precedent of Tat-based DNA vaccines in conferring some level of protection in animal models, we believe that the virologic control demonstrated with this novel multimerised Tat vaccine highlights the promise of this vaccine candidate for humans.

The HIV Tat protein is required for efficient virus replication^1^,^2^ and is released into the extracellular milieu by infected cells where it can be taken up by other cells to increase transcription from the HIV long terminal repeat (LTR)^1^. The *tat* gene is more genetically stable than the *env* gene and immunogenic Tat epitopes are conserved across HIV-1 subtypes in group M^3^. Thus, a Tat vaccine may provide cross protection across all group M viruses, which account for a majority of HIV infections globally^4^. These attributes make Tat a potential component of an HIV vaccine. As it has been difficult to generate canonical Env-specific neutralising antibodies (NAb)^5^, the production of high titre anti-Tat NAb might be a feasible alternative to control HIV replication and delay disease onset. Tat-based HIV vaccines are safe and generate high titre Tat-specific humoral and cell mediated immunity (CMI) that correlate with asymptomatic infection or slower disease progression in humans^6^,^7^ and animals. However, native Tat is poorly immunogenic, easily oxidised and degraded by proteolysis^1^. Thus, protecting Tat from these detrimental processes improves the immunogenicity of Tat-based vaccines^8^,^9^. We now report a novel strategy that incorporates Tat into heptamers by fusing Tat with the oligomerisation domain of the C4 binding protein (C4b-p), an inhibitor of the classical and lectin pathways of complement^10^. Previously the most effective form, termed IMX313, a hybrid derived from the oligomerisation domains of human and murine C4b-p α-chains^11^,^12^,^13^ induced oligomerisation of *P. falciparum* or *M. tuberculosis* antigens and enhanced the immunogenicity and protective efficacy of the resultant vaccines^11^,^12^,^13^,^14^. The IMX313 amino acid sequence is <20% identical to the murine or human C4b-p oligomerisation domains, thus IMX313 does not induce antibodies^12^, and has recently been shown to be safe for use in humans^15^ making it a practical molecular adjuvant. However, as the adjuvanticity of IMX313 requires efficient secretion of the oligomerised protein^14^, a TPA leader sequence was introduced upstream of the sequences encoding Tat and IMX313 to drive expression of the oligomerised protein into the secretory pathway. Indeed, DNA and recombinant viral vaccines encoding IMX313 are in advanced clinical testing^16^. DNA vaccines are stable, readily manufactured, induce humoral and CMI, and are licensed for veterinary use^17^, emphasising their potential in developing human vaccines. To improve the immunogenicity of Tat-based DNA vaccines, we fused Tat to IMX313, and compared the immunogenicity and protective efficacy of this vaccine with that of the corresponding vaccines lacking IMX313 with a major thrust to elicit robust humoral responses.

## Results

### Tat oligomerisation

**Figure 1:**
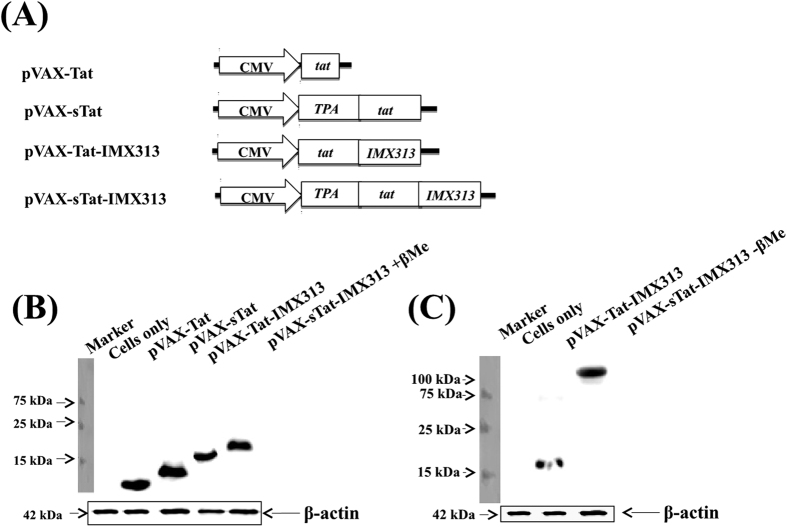
Vaccine constructs and Tat expression. (**A**) Schematic representation of the vaccine constructs; (**B**) reducing Western blot analysis of Tat in cell lysates from HEK293T cells transfected with plasmid DNA encoding the different forms of Tat (tracks 2–5) and (**C**) non-reducing Western blot analysis of Tat in supernatant fluids of HEK293T cells transfected with pVAX-Tat-IMX313 (track 2) or pVAX-sTat-IMX313 (track 3) DNA. The 42 kDa β-actin protein was used as loading control for the Western blot. Blots in Fig. 1B,C are cropped for clarity and conciseness; full length blots of these figures are presented in supplementary figure S1A,B.

Four plasmids, each encoding a different form of HIV Tat were constructed (Fig. 1A). In this study, the DNA vaccine encoding the native form of Tat protein was used as the benchmark against which the efficacy of plasmids encoding secreted and/or oligomerised forms of Tat were compared. Native Tat antigen, was not used as a control, as others have shown that its immunogenicity is affected due to its susceptibility to oxidation and proteolysis^1^. To confirm expression of Tat, HEK293T cells were transfected with plasmid DNA, and Western blot analysis performed on all four cell lysates (Fig. 1B) and supernatant fluids from pVAX-Tat-IMX313 or pVAX-sTat-IMX313-transfected cells (Fig. 1C). Oligomers of protein-IMX313 fusions can only be detected in non-reducing conditions^12^,^14^ and consequently in a reducing Western blot, 11, 14, 18 and 20 kDa bands corresponding to proteins expressed from pVAX-Tat, pVAX-sTat, pVAX-Tat-IMX313 and pVAX-sTat-IMX313, respectively, were detected (Fig. 1B) and supplementary figure S1A (for full size blot).The assay was repeated in the absence of β-Me and bands of ~18 kDa from pVAX-Tat-IMX313-and ~140 kDa from pVAX-sTat-IMX313-transfected cells detected (Fig. 1C) and supplementary figure S1B (for full length blot). Thus the different forms of Tat were detected as predicted and fusion of IMX313 to sTat resulted in Tat oligomerisation. However, as oligomerisation requires secretion of the fusion protein^14^, expression from pVAX-Tat-IMX313 probably failed to result in an oligomer of~126 kDa under non-reducing condition (Fig. 1C), due to cytoplasmic retention of the protein.

### DNA vaccines encoding Tat generate T-cell immune responses *in vivo*

**Figure 2:**
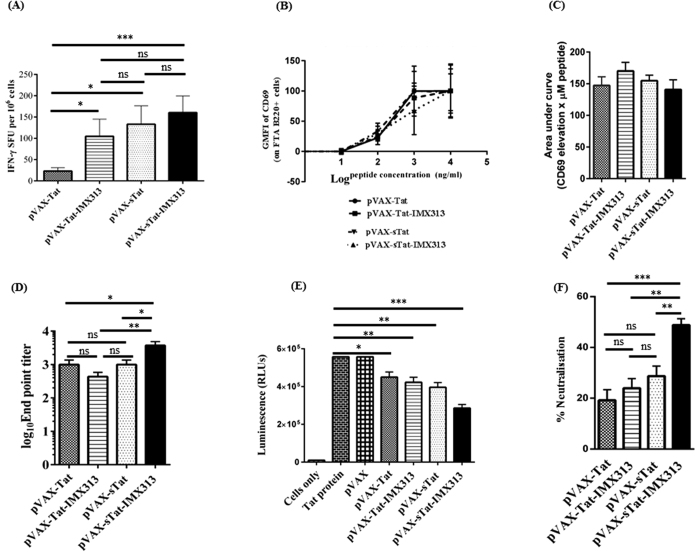
Tat DNA vaccination induces Th cell responses and humoral immunity. Mice (n = 7 per group) were vaccinated ID with 3 doses of 50 μg of pVAX, pVAX-Tat, pVAX-Tat-IMX313, pVAX-sTat or pVAX-sTat-IMX313 at 2 week intervals. Splenocytes were collected 14 days after the last dose. Blood was collected one day before each vaccination and on the day of euthanasia. (**A**) IFN-γ ELIspot assay to examine Tat-specific CMI in splenocytes stimulated with Tat peptides for 36 h. (**B**) FTA assay to examine Th cell responses from pVAX-Tat, pVAX-Tat-IMX313, pVAX-sTat or pVAX-sTat-IMX313 vaccinated mice above the GMFI of CD69 on FTA B220^+^ cells from naïve mice. The graph is representative of the FTA with cells pulsed with Tat peptide pools 1, 2 or 3. (**C**) AUC values for Th cell responses depicted in (**B**). (**D**) The results of an indirect ELISA to detect Tat-specific serum IgG. Titres are expressed as the reciprocal of the serum dilution and plotted as log10 IgG endpoint titre. The data are representative of 2 independent experiments in which each serum sample was analysed in duplicate. (**E**) Tat transactivation activity in Cf2-Luc cells after the addition of Tat pre-incubated in PBS or a 1/25 dilution of serum samples from mice vaccinated with the respective vaccines. Graphs show mean RLUs (±SEM) relative to untransfected control cells and are representative of 3 independent experiments in which each sample was analysed in triplicate. (**F**) Percentage decline in luminescence (an indicator of the neutralisation of Tat transactivation activity) depicted in (**E**). Data shown in the entire figure depict the mean (n = 7) ± SEM; an unpaired non-parametric Mann–Whitney U test was used to analyse the statistical significance of the data; *p < 0.05, **p ≤ 0.01, ***p ≤ 0.001 and *p* ≥ 0.005 = non-significant (ns).

Tat-specific CMI is associated with improved virological control in humans^1^ and non-human primates^18^,^19^. Initially, we evaluated the ability of the DNA vaccines to induce CMI using IFN-γ ELISpot as a surrogate indicator of Tat-specific CMI in vaccinated animals. The animals received 3 vaccine doses, and splenocytes harvested 14 days later were re-stimulated for 36 h with Tat peptides which included the Tat epitopes targeted by CTL in BALB/C mice^20^. Responses were significantly higher in splenocytes from pVAX-Tat-IMX313 (mean SFU of 105, *p* = 0.0332), pVAX-sTat (133, *p* = 0.0239) and pVAX-sTat-IMX313 (160, *p* = 0.0006)-vaccinated mice than pVAX-Tat-vaccinated animals (23) (Fig. 2A). pVAX-sTat-IMX313 generated slightly higher responses than pVAX-Tat-IMX313 (*p* = 0.197) or pVAX-sTat (*p* = 0.05134). These results suggest that Tat immunogenicity was enhanced when Tat was secreted and fused to IMX313.

We also measured the vaccine-induced CMI by CTL activity and T-helper (Th) cell responses *in vivo* using the fluorescent target array (FTA) assay^21^. The FTA target cells were injected into vaccinated animals 13 days after the third vaccine dose, the splenocytes harvested 18h later and analysed by multi-parameter flow cytometry for CTL and Th responses *in vivo*. Initially, CTL activity was examined and showed <1% killing of peptide-pulsed FTA-target cells, a level of killing that was similar across all vaccinated groups (Supplementary Fig. S2B). This was unexpected and contrasts with previous studies reporting potent CTL activity post Tat vaccination^1^,^22^.

Second, we examined Th responses mediated by CD4^+^Th cells required for efficient maturation and activation of B cells^23^. The CD4^+^Th cell response was determined by measuring the up-regulation of CD69 on peptide-pulsed B cells in the FTA of challenged mice, relative to controls. The data showed that there was a dose-dependent elevation of CD69 expression on FTA B cells in all vaccinated animals (Fig. 2B) but no significant difference between the different groups. To assess the cumulative magnitude of Th cell responses induced following vaccination, we calculated the area under the curve (AUC) for the data analysed in Fig. 2B, but this analysis also revealed similar Th cell responses in all vaccinated groups (Fig. 2C). Collectively, these results suggest that pVAX-Tat, pVAX-Tat-IMX313, pVAX-sTat and pVAX-sTat-IMX313 elicited Th cell responses capable of activating naïve B cells, but were ineffective in generating detectable CTL responses.

### DNA vaccines encoding Tat induce anti-Tat NAb

As Tat-specific serum IgG capable of neutralizing Tat activity is desirable for an effective Tat-based vaccine^7^,^8^,^24^, we analysed serum samples from vaccinated mice for Tat-specific antibodies by ELISA. Tat-specific IgG was detected in serum with mean titres of 433 for pVAX-Tat-IMX313, 1000 for pVAX-Tat, 1029 for pVAX-sTat and 3728 for pVAX-sTat-IMX313 (Fig. 2D). Thus, pVAX-sTat-IMX313 elicited an antibody titre 9-fold higher than that elicited by pVAX-Tat-IMX313 and ~3.6-fold higher than that from pVAX-Tat or pVAX-sTat. These results also demonstrated that Tat immunogenicity was enhanced when the DNA vaccine encoded a secreted and oligomerised version of Tat (pVAXsTat-IMX313).

We next examined whether anti-Tat-specific antibodies could neutralize Tat transactivation activity *in vitro*. Tat protein pre-incubated in PBS or in a set dilution of serum (1/25) from vaccinated mice was added to Cf2 cells. It is expected that once the Tat protein is added to Cf2 cells, it will be taken-up by the cells and up-regulate transcription from the HIV LTR resulting in expression of luciferase and thus luminescence. However, Tat-specific antibodies that are present in the serum of vaccinated animals will bind and neutralize Tat thereby blocking its uptake into cells and inhibiting its transactivation activity, resulting in reduced or no luminescence. The mean RLU in cells incubated with Tat in PBS was 557345 (Fig. 2E). A similar result was obtained from cells to which the Tat protein pre-incubated with diluted serum from pVAX-vaccinated mice was added. Importantly pre-incubation of Tat protein with diluted serum samples from the Tat-vaccinated mice, resulted in a clear reduction of mean RLUs. Mice vaccinated with pVAX-Tat showed a 19% decline (mean RLU 451449), pVAX-Tat-IMX313 a 24% decline (mean 423582), pVAX-sTat a 29% decline (RLU 395715) while pVAX-sTat-IMX313 vaccinated mice showed the most significant reduction of 49% (mean 285758) (Fig. 2E,F). This level of neutralisation appeared to correlate with the previously determined anti-Tat antibody titre. As the pVAX-sTat-IMX313 and pVAX-sTat vaccines induced the strongest anti-Tat NAb, they were selected for further analysis.

### Higher titre anti-Tat responses were induced by multiple vaccine doses

**Figure 3:**
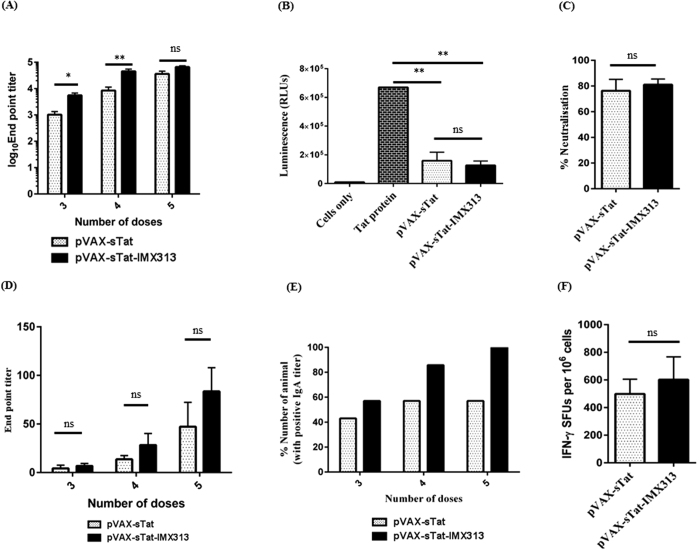
Humoral responses and CMI are increased after 5 doses of Tat DNA vaccine. Animals were vaccinated ID with 5 doses of 50 μg of pVAX-sTat or pVAX-sTat-IMX313 at 2 week intervals. Splenocytes were collected 14 days after the last dose. Blood and CVLs were collected one day before each vaccination and on the day of euthanasia. (**A**) ELISA results showing serum anti-Tat IgG titres. (**B**) Serum neutralisation of Tat transactivation activity, (**C**) Percentage decline in luminescence (an indicator of the neutralisation of Tat transactivation activity) that is depicted in (**B,D**) ELISA results showing anti-Tat sIgA titres in CVLs from pVAX-sTat or pVAX-sTat-IMX313 vaccinated mice. Data are representative of 2 independent experiments. (**E**) Percentage of vaccinated animals in which sIgA was detected after 3–5 vaccine doses in the experiment shown in 3D. (**F**) IFN-γ ELIspot assay to examine Tat-specific CMI responses following administration of 5 doses of pVAX-sTat or pVAX-sTat-IMX313. Data shown in the entire figure depict the mean (n = 7) ± SEM; an unpaired non-parametric Mann–Whitney U test was used to analyse the statistical significance of the data; *p < 0.05, **p ≤ 0.01, ***p ≤ 0.001 and *p* ≥ 0.005 = non-significant (ns).

High titre anti-Tat antibodies develop after multiple vaccine doses^7^,^18^,^19^,^25^ thus, we determined if an increased number of doses of pVAX-sTat or pVAX-sTat-IMX313 would the antibody titres. Consequently, mice were vaccinated with a total of 5 doses of 50 μg DNA, and serum and CVL samples examined for anti-Tat IgG and mucosal IgA, respectively, in samples collected after the 3^rd^, 4^th^ and 5^th^ doses. In addition, splenocytes were harvested and analysed by ELISpot. After the 4^th^ dose, serum anti-Tat IgG titres increased by ~4.7 fold in all pVAX-sTat vaccinated mice, from a mean endpoint titre of 1800 to 8486 (*p* = 0.0262) and by ~8.16 fold in pVAX-sTat-IMX313, from a mean endpoint 5526 to 45129 (*p* = 0.0006) (Fig. 3A). Significantly, inclusion of IMX313 resulted in an increase of ~5-fold in the titres from pVAX-sTat-IMX313 mice compared to pVAX-sTat-vaccinated mice (*p* = 0.0029). Administration of a 5^th^ dose increased the serum IgG titres by ~4 fold (8486 to 35871, *p* = 0.0152) in pVAX-sTat- and by ~1.5 fold (45129 to 65957, *p* = 0.2657) in pVAX-sTat-IMX313-vaccinated animals (Fig. 3A). At this time point, the difference in serum titres between the two groups was no longer significant (*p* = 0.0862).

To assess if the higher titres also resulted in increased NAb activity, we repeated the Tat transcription assay using serum collected 2 weeks after the 5^th^ vaccine dose. This showed an increase in Tat neutralisation activity of 4.09 fold (i.e. reduced RLUs from 488049 to 119040 RLUs, 27% to 76% neutralisation, *p* = 0.0043) for pVAX-sTat and by 2.68 fold (from 341062 to 127144, 49% to 81% neutralisation, *p* = 0.0022) for pVAX-sTat-IMX313 relative to the samples taken after 3 doses (compare Fig. 3B,C with 2E,F). However, the difference in percentage neutralisation between the two vaccines at this time point was not significant (*p* = 0.7879).

### The Tat DNA vaccine induced sIgA responses in CVL

We tested CVL samples for sIgA as anti-Tat sIgA could potentially block HIV activity at the mucosa^26^,^27^. Low levels of sIgA were detected in 42% (3/7) pVAX-sTat- and 57% (4/7)pVAX-sTat-IMX313-vaccinated mice after the 3^rd^ dose (mean IgA titres of 4 and 7, respectively) (Fig. 3D,E). After administration of the 4^th^ dose, sIgA was detected in 57% (4/7) pVAX-sTat and 85% (6/7) pVAX-sTat-IMX313 mice, while the mean titres increased by ~3.5-(*p* = 0.054) and 4-fold (*p* = 0.0431) to 14 and 28 respectively (Fig. 3D,E). Importantly, after the 5th dose, sIgA was detected in 100% (7/7) of pVAX-sTat-IMX313 mice, while it had no effect on pVAX-sTat vaccinated mice (57%; 4/7). At this time point, the mean sIgA titres increased by ~3 fold for each vaccine and reached 47 (*p* = 0.554) and 84 (*p* = 0.554) in samples from pVAX-sTat and pVAX-sTat-IMX313 vaccinated animals, respectively (Fig. 3D,E). The difference in titres was not significant, although only pVAX-sTat-IMX313 induced Tat-specific sIgA in all vaccinated animals.

### Increasing vaccine doses enhances CMI to Tat

We then examined CMI to Tat by ELIspot 2 weeks after 5 doses of pVAX-sTat or pVAX-sTat-IMX313. Lower responses were detected in splenocytes from pVAX-sTat than pVAX-sTat-IMX313-vaccinated mice (mean SFUs 498 vs 601, *p* = 0.7768), but this difference was not statistically significant (Fig. 3F). These responses were ~4 fold higher than those generated after 3 doses of pVAX-sTat-IMX313 (total mean IFN-γ SFUs 601 vs 160, *p* = 0.0163) or pVAX-sTat (total mean IFN-γ SFUs 498 vs 133, *p* = 0.0163) suggesting that administration of 2 extra booster doses enhanced CMI. However, a caveat for these results is that the comparison between CMI generated after the 3^rd^ and 5^th^ doses is based on results from 2 different experiments.

### DNA vaccines encoding Tat control EcoHIV viral load post-challenge

**Figure 4:**
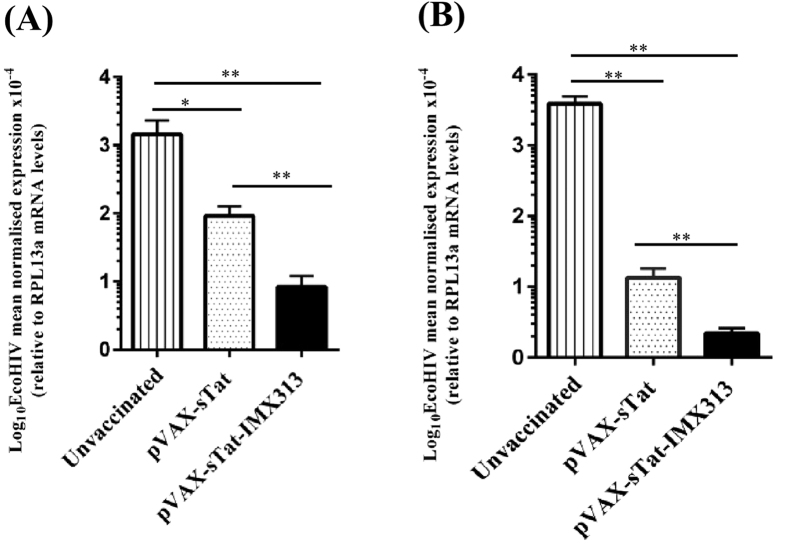
pVAX-sTat-IMX313 vaccinated mice exhibit superior control against EcoHIV challenge. Unvaccinated mice or vaccinated mice which received 5 doses of 50 μg of either pVAX-sTat or pVAX-sTat-IMX313 were challenged with 1.5 μg p24 of EcoHIV/NL4-3 10 days post final vaccination. The viral load in PECs and splenocytes was determined by qRT-PCR; EcoHIV RNA levels in (**A**) PECs and (**B**) splenocytes 7 days post challenge. EcoHIV mRNA levels were normalised to RPL13a mRNA and the data represent the mean (n = 7) ± SEM. **p < 0.05 and **p ≤ 0.01(Mann–Whitney U test).

To the best of our knowledge, EcoHIV is the only surrogate murine HIV challenge model and has been used previously to successfully evaluate the efficacy of candidate HIV vaccines^28^,^29^. There is no documented evidence that EcoHIV can infect mice via the intravaginal route which accounts for a majority of HIV transmission^30^ and thus the intraperitoneal route has been used in our laboratory and by other research groups as a convenient route to successfully deliver the virus^28^,^29^,^31^,^32^. Furthermore, mice are infected following a single EcoHIV challenge resulting in spread of the infection from the primary infection site to the spleen and the brain in a manner reminiscent of HIV infection^33^. EcoHIV encodes the complete range of HIV proteins, including Tat, from the HIV-1 Clade B NL4-3 strain, except for the HIV gp120 which was replaced with gp80 from the murine leukemia virus^33^. Consequently, as EcoHIV infects murine lymphocytes^33^, we examined the protective efficacy of vaccination with pVAX-sTat or pVAX-sTat-IMX313 against EcoHIV challenge in mice which received 5 doses of the vaccines. The level of EcoHIV infection was determined by measuring the viral load in PECs and splenocytes from challenged animals by RT-qPCR targeting the MLV *env* gene and the results were normalised to the RPL13a house-keeping gene as described previously^28^,^29^. However, no clinical data eg. CD4^+^ cell counts, were gathered as the primary site of infection is peritoneal cells and because the virus causes no overt disease^33^. Both groups of vaccinated mice showed significantly reduced levels of EcoHIV RNA in PECS and splenocytes compared with unvaccinated mice. The viral load in pVAX-sTat-IMX313 vaccinated mice was reduced by ~11 fold in PECS (*p* = 0.0022) and ~7 fold in splenocytes (*p* = *0.*0022) compared with pVAX-sTat vaccinated mice (Fig. 4A,B) although pVAX-sTat- and pVAX-sTat-IMX313-vaccinated mice limited EcoHIV infection significantly compared to unvaccinated control mice (*p* = 0.0012 and *p* = 0.0022, respectively). Overall, the data show that oligomerisation of a secreted form of Tat can significantly augment anti-Tat mucosal and systemic antibody responses, and importantly increases the protective efficacy of Tat DNA vaccines.

## Discussion

In this study, we exploited a novel strategy to generate high titre Tat-specific serum IgG with potent anti-Tat neutralisation activity following administration of a DNA vaccine encoding sTat fused to the oligomerisation domain of C4b-p (pVAX-sTat-IMX313). The vaccines were administered via the ID route as this site is rich in antigen presenting cells^34^,^35^. pVAX-sTat-IMX313 elicited high titre Tat-specific systemic IgG and mucosal sIgA in all vaccinated mice and significantly reduced the EcoHIV viral load compared to unvaccinated controls, but surprisingly only resulted in a modest cell-mediated response. Although the mechanism of C4b-p induced protein oligomerisation is not well defined^12^,^14^, our results suggest that oligomerisation requires efficient secretion of the fusion protein (Fig. 1B,C), consistent with previous studies^11^,^12^,^14^.

Although oligomerised proteins are more stable, resistant to oxidation and proteolysis^8^,^9^, and more immunogenic compared to their monomeric counterparts, canonical Tat protein-based vaccines are poorly immunogenic, likely due to the poor stability of native Tat protein^1^,^2^. A few studies suggest that an increase in Tat stability and protection from proteolysis is required to enhance Tat immunogenicity^8^,^9^. Consequently, our studies were restricted to a comparison of different DNA constructs. Indeed, pVAX-sTat-IMX313 was superior in inducing anti-Tat NAb responses compared to the other DNA vaccines tested. The appearance of anti-Tat antibodies appears to correlate with protection in humans^6^,^7^,^24^,^36^,^37^ and vaccination with DNA encoding Tat induced protection in non-human primates^18^,^22^,^25^ thus, inducing high titre Tat-specific antibodies is an important component of future HIV vaccines as these antibodies are expected to neutralise Tat activities thereby blocking the early stages of virus replication.

Tat-based vaccines may not be expected to elicit sterilizing immunity, but to control HIV replication and disease onset^1^, and control of virus burden normally requires potent CTL responses^18^,^38^. Current assays which evaluate T cell immunity (eg. ELISpot, ICS, and ^51^Cr release) are performed *ex vivo* after *in vitro* stimulation of T cells. Whilst these assays provide useful information, they involve extensive (several hours to days) manipulation of T cells *in vitro* and are not directly indicative of T cell killing function *in vivo*. The FTA is currently the most sensitive and versatile assay available to evaluate CD4^+^ and CD8^+^ T cell effector functions *in vivo* in preclinical models^21^. The assay essentially involves ‘barcoding’ targets with a combination of cell tracking dyes (eg. CFSE, Cell Proliferation Dye eFluor670 (CPD) and Cell Trace Violet (CTV)) to generate up to 252 unique cell clusters as targets that can be pulsed with various peptides prior to FTA challenge of vaccinated animals^21^. This technique allows for sensitive and direct measurement of *in vivo* killing in vaccinated animals, as well as for the measurement of CD4^+^ T helper cell activity based on their ability to up-regulate CD69 expression. Thus, we examined *in vivo* whether the Tat DNA vaccines induced a CTL response in vaccinated animals using this assay. Surprisingly, the FTA killing assay revealed <1% killing of target cells. This result was in clear contrast to previous studies which reported potent CTL responses after Tat vaccination^18^,^22^. It is likely that CTL responses are affected by the route and dose of each vaccine, as suggested in other previous studies^18^,^25^. However, using the same assay we observed an equally potent Th response *in vivo* in all Tat DNA vaccinated mice (Fig. 2C). Based on these results, we anticipated that similar levels of anti-Tat antibodies should be generated following vaccination with pVAX-Tat, pVAX-Tat-IMX313, pVAX-sTat or pVAX-sTat-IMX313. Interestingly, the highest antibody titre and serum neutralisation was detected in serum from pVAX-sTat-IMX313 vaccinated mice suggesting that the generation of these antibodies was not exclusively dependent on the magnitude of T-cell help. Whether the improved stability and oligomerisation of secreted Tat resulted in greater antigen presentation to B cells independently of CD4^+^ T cells or potent cross-linking of the B-cell receptor was not explored in this study and warrants further investigation.

Tat vaccines often require >3 doses to induce robust immune responses^7^,^18^,^22^,^39^ and our data showed that two additional doses significantly enhanced Tat IgG titres and NAb responses, to neutralise 76 and 81% Tat activity activity for pVAX-sTat and pVAX-sTat-IMX313, respectively (Fig. 3C), although the differences were not statistically significant. As the concentration of Tat protein (50 ng/ml) added to the transactivation assay is similar to that of soluble Tat detected in serum from HIV-infected individuals^40^ and was largely neutralised by a 1/25 dilution of mouse serum, a similar or greater level of neutralisation of Tat transcription activity would be expected following administration of pVAX-sTat-IMX313 in humans.

Furthermore, 100% of pVAX-sTat-IMX313-vaccinated mice but only 57% of pVAX-sTat-vaccinated mice (Fig. 3E) developed mucosal sIgA proving that pVAX-sTat-IMX313 generated mucosal humoral responses more effectively. The presence of sIgA at the mucosa and in blood appears to correlate with protection against HIV in exposed, persistently seronegative individuals^41^,^42^,^43^ and in non-human primates^44^,^45^. Since the mucosa is the site for primary HIV infection^46^, and humoral responses at the mucosa are mainly mediated by sIgA^47^, we expect the presence of Tat-specific sIgA at mucosal site to confer similar protective benefits in pVAX-sTat-IMX313-vaccinated individuals. In addition to boosting humoral responses, increasing the number of doses to five significantly increased Tat-specific CMI, with pVAX-sTat-IMX313eliciting slightly higher CMI than pVAX-sTat (Fig. 3).We were unable to perform the FTA on animals which received five doses to determine if this would result in increased CTL killing and ongoing experiments are planned to address this issue.

Robust immune responses are not necessarily indicative of protection against virus challenge^48^,^49^. Therefore, an effective Tat-based HIV vaccine should demonstrate efficacy against viral replication and control disease progression. To investigate this aspect, we challenged pVAX-sTat-IMX313 and pVAX-sTat vaccinated mice with EcoHIV. The results from this experiment showed that the pVAX-sTat-IMX313 vaccine elicited superior control of virus replication at the site of infection (peritoneal space) and control over EcoHIV spread to the spleen compared with pVAX-sTat vaccination (Fig. 4A,B). However, we did not investigate the longevity/durability of EcoHIV control in vaccinated animal. Experiments to address these aspects will require a non-human primate model. It is unclear if humoral immunity and/or CMI was responsible for the observed control of EcoHIV infection as we were unable to conduct a correlation analysis owing to the limited number of animals studied per vaccination group. As there is no evidence for an interaction between the MLV envelope and HIV Tat, anti-Tat antibodies are unable to bind to- or inhibit uptake of-EcoHIV. Therefore, based on the *in-vitro* Tat neutralisation data presented here, we are tempted to speculate that the control of the EcoHIV viral load was associated with the presence of anti-Tat neutralising antibodies as reported in previous animal studies^50^,^51^,^52^, but we cannot discount the potential of CMI to control the viremia and an additional study to address this is warranted. Although we did not determine if the mice generated anti-IMX313 antibodies, a previous^12^ noted that such antibodies were not detected. Irrespective of this, the Tat antigen used in the transactivation assay did not contain the IMX313 sequence and thus the neutralisation was specific for Tat. In addition, although anti-Tat antibodies may contribute to ADCC^53^, we did not investigate whether the antibodies had ADCC activity.

As there was no statistical difference in antibody titres after 5 doses of pVAX-sTat-IMX313 or pVAX-sTat vaccination (Fig. 3A), the EcoHIV challenge data also suggest that pVAX-sTAT-IMX313 elicited antibodies of superior quality compared with pVAX-sTat.

Collectively, the results show that pVAX-sTat-IMX313 was most immunogenic and compared favourably with previous Tat-based vaccines^7^,^18^,^22^. The ability of pVAX-sTat-IMX313 to induce high titre anti-Tat responses can most likely be attributed to Tat oligomerisation and the secretion of Tat heptamers as particulate antigens such as oligomerised proteins, lead to greater uptake by phagocytosis and result in enhanced antibody production^12^,^14^. Previously, the formation of plasmodium merozoite surface proteins and *Mycobacterium tuberculosis* antigen 85A heptamers enhanced the immunogenicity of candidate malaria^11^,^12^,^14^ and tuberculosis vaccines^13^ respectively. Although the mechanism has not been defined, it has been suggested that IMX313 binds to B-cell CD40, C-reactive proteins, pentraxins and serum amyloid P-complexes resulting in increased Fcγ-receptor-mediated phagocytosis and cytokine secretion^14^. This interaction also enhances CD54 (ICAM-1), CD86 expression and IL4-dependent IgE isotype switch^54^ resulting in increased antigen presentation by APCs^13^,^55^ and prolongation of serum half-life of the immunogens^12^,^56^,^57^. Consequently, these interactions enhance adaptive immune responses^14^ which might explain why the pVAX-sTat-IMX313 vaccine induced high titre anti-Tat responses. Thus, IMX313 acts as a molecular adjuvant when included in DNA vaccine formulations.

For optimal efficacy, the pVAX-sTat-IMX313 vaccine could be combined with other DNA vaccines, and as we previously reported that a unique cytolytic DNA-gag vaccine also elicits significant control of EcoHIV infection post-challenge^28^, experiments to address this are planned. Our results support further testing of pVAX-sTat-IMX313 as a candidate HIV vaccine in a larger animal model and avoid the need to purify Tat, thus circumventing the disadvantages associated with proteolytically degraded and oxidised Tat protein.

## Materials and Methods

### DNA plasmids

Codon optimised HIV-1 clade B *tat* and IMX313 genes (Gene Art, Germany) were inserted into pVAX downstream of the CMV promoter. Four plasmids were generated encoding either wild type Tat viz. (1) pVAX-Tat, (2) pVAX-Tat-IMX313 or secreted Tat (sTat) generated by the upstream introduction of the TPA leader sequence^29^ (3) pVAX-sTat and (4) pVAX-sTat-IMX313. pET-DEST42, encoding Tat fused to a 6x-His tag was used to synthesize the protein in *Escherichia coli* for ELISA. PCR primers for cloning are listed in the supplementary material and all plasmids were sequenced. DNA vaccines were purified using standard molecular biology and endotoxins removed with the endotoxin removal solution (Sigma-Aldrich, Sydney, Australia). The endotoxin level in each DNA vaccine was <0.1 EU/μg DNA (SA Pathology, Adelaide, Australia).

### Western blot analysis

To detect Tat expression, cell lysates and supernatants were harvested from HEK293T cells transfected with DNA and 50 μg of protein was analysed in 10–12% (v/v) SDS-PAGE under reducing (with β-mercaptoethanol, β-Me) or non-reducing (without β-Me) conditions as described^12^,^14^. A cocktail of Tat monoclonal antibodies, 4D5.24, 5A5.3 and 1D9 (NIH AIDS Reagent Program) and goat-anti mouse HRP-conjugated secondary antibody (Antibodies Australia, VIC, Australia) were used to detect Tat expression essentially as described previously^29^.

### Animal immunisations

Female 6–8 week old BALB/c mice purchased from the University of Adelaide Animal Services were maintained in the Queen Elizabeth Hospital animal house under PC2 conditions in individually ventilated cages fitted with a HEPA filter. All experiments were approved by and conducted in accordance with guidelines and protocols approved by the University of Adelaide and the South Australia Pathology Animal Ethics Committees. Groups (n = 7) were vaccinated with the different vaccines or pVAX (n = 3) with 3–5 doses of 50 μg in saline at 2 week intervals, by the ID route as described^28^,^29^. Blood and cervical vaginal lavage (CVL) samples were collected^28^ and examined before and after each vaccine dose for anti-Tat antibody responses; 14 days post vaccination, the mice were euthanized and splenocytes prepared for analysis.

### Enzyme-linked immunosorbent spot assay (ELISpot)

Splenocytes were stimulated with a panel of 11 overlapping 15–19 mer peptides (NIHAIDS reagent program) spanning the entire Tat proteinas we described^28^,^29^. Briefly, multiscreen-IP HTS plates (Merck Millipore, Germany) were coated with anti-mouse IFN-γ (clone AN18, MabTech, Sweden) and secreted IFN-γ detected with anti-mouse IFN-γ -biotin (clone R4-6A2, MabTech).Phytohemagglutinin and un-stimulated splenocytes were used as positive and negative controls, respectively. Spots were counted using an ELISpot reader (AID GmbH, Germany). The number of spots in unstimulated splenocytes was subtracted from the number in peptide-stimulated cells to generate the net number of Tat-specific spot forming units (SFUs).

### Fluorescent target array (FTA) assay

The FTA assay was used to examine the magnitude and quality of T-cell responses generated after vaccination in an *in vivo* assay^21^. Splenocytes from naïve BALB/c mice were co-labelled with combinations of four different concentrations of cell trace violet (CTV) and carboxyfluoresceinsuccinimidyl ester (CFSE) dyes to generate a 16 parameter FTA (Supplementary Fig. S2A), as described previously^21^.

The fluorescent tagged cells were then pulsed with 0.01–10 μg/ml of Tat peptides for 4 h at 37 °C in 5% CO_2_. The peptides were divided into pools 1–3 in which pool 1 contained peptides representing the complete Tat protein, whereas pool 2 lacked the theoretical CD8^+^ T cell immunodominant epitopes and pool 3 contained only the theoretical CD8^+^ T cell immunodominant epitopes (HIV molecular immunology database, http://www.hiv.lanl.gov/). The peptide-pulsed dye-labelled cells were pooled and about 40 × 10^6^ cells were adoptively transferred intravenously via the lateral tail vein into each mouse, 13 days after the final vaccine dose. Splenocytes were then harvested 18h post-FTA transfer, RBC-depleted and stained with antibodies against mouse antigens (allophycocyanin conjugated anti-CD69 (eBioscience) and Alexa Fluor 700 conjugated anti-B220 (eBioscience)). The percentage specific killing of FTA cells was calculated using the formula described previously^24^. The magnitude of CD4^+^ T cell help provided to naïve B cells in vaccinated mice was determined by measuring the geometric mean fluorescence intensity (GMFI) of CD69 on gated B220^+^ cells within the FTA. Samples from each vaccinated mouse were analysed on a BD FACS Canto (Becton Dickinson, NJ, USA) and FACS plots and GMFI analyses were performed using the FlowJo software (version 8.8.7).

### Enzyme-linked immunosorbent assay (ELISA)

Serum and CVL samples were analysed for anti-Tat antibodies by indirect ELISA. Briefly, Maxisorp plates (Corning Sigma-Aldrich) were coated with 500 ng of Tat, then serum and CVL samples (diluted in PBS/0.5% FCS) added. Bound antibodies were detected using HRP-conjugated goat anti-mouse IgG (GE Healthcare Life Sciences, USA) or anti-mouse IgA (LifeTechnologies) and the OD read at 492 nm. Endpoint titres were determined as the reciprocal of the highest serum or CVL sample dilution with an OD reading above the cut-off, set as 2SD above the mean OD of serum samples from pre-vaccinated- or naïve- mice.

### Anti-Tat neutralisation assay

The NAb activity of anti-Tat antibodies was assessed in an *in vitro* transactivation assay using Cf2-Luc cells^3^,^58^. 50 ng/ml of purified Tat protein (NIH Reagent Bank), previously incubated for 1h in PBS or serum diluted 1/25, was added to the cells, and cell lysates prepared 48 h later as described^58^,^59^ then assayed for luciferase activity using the dual luciferase assay system (Promega). The data are expressed as mean relative luminescence units (RLUs) ± SEM. The percentage neutralisation was calculated using the formula: % specific neutralisation = [(RLUs in the absence of test sera- RLUs in the presence of test sera)/ RLUs in the absence of test sera] x100.

### EcoHIV/NL4-3 challenge

EcoHIV was prepared and titrated as described^28^,^29^ and vaccinated mice challenged via the intra-peritoneal route with EcoHIV equivalent to 1.5 μg p24^28^,^29^. The mice were culled, and spleen and peritoneal exudate cells (PECs) collected 7 days post challenge then examined by qRT-PCR^28^,^29^.

### Statistical analysis

Data presented as mean ± SEM were generated with GraphPad Prism version 6 (GraphPad Software, La Jolla, CA, USA). Non-parametric Kruskal-Wallis test was used to compare the difference between multiple vaccine groups. If this showed significant differences, then the Mann-Whitney U test was performed to compare differences between each vaccine group, independently. Statistical significance was determined using the Mann-Whitney U test; *p* < 0.05 was considered significant and *p* > 0.05 was considered non-significant.

## Additional Information

**How to cite this article**: Tomusange, K. *et al*. A HIV-Tat/C4-binding protein chimera encoded by a DNA vaccine is highly immunogenic and contains acute EcoHIV infection in mice. *Sci. Rep.* **6**, 29131; doi: 10.1038/srep29131 (2016).

## Supplementary Material

Supplementary Information
