# Supplementary material for: A HIV-Tat/C4-binding protein chimera encoded by a DNA vaccine is highly immunogenic and contains acute EcoHIV infection in mice
Source: Sci Rep. 2016 Jun 30;6:29131. doi: 10.1038/srep29131 (PMC4928126; doi:10.1038/srep29131)
Supplement: Supplementary Information [file srep29131-s1.pdf]

A HIV-Tat/C4-binding protein chimera encoded by a DNA vaccine is highly immunogenic and contains acute EcoHIV infection in mice

Khamis Tomusange[1], Danushka Wijesundara[1], Jason Gummow[1], Tamsin Garrod[2], YanruiLi1, Lachlan Gray[3, 4], Melissa Churchill[3], Branka Grubor-Bauk[1] and Eric J. Gowans[1#]

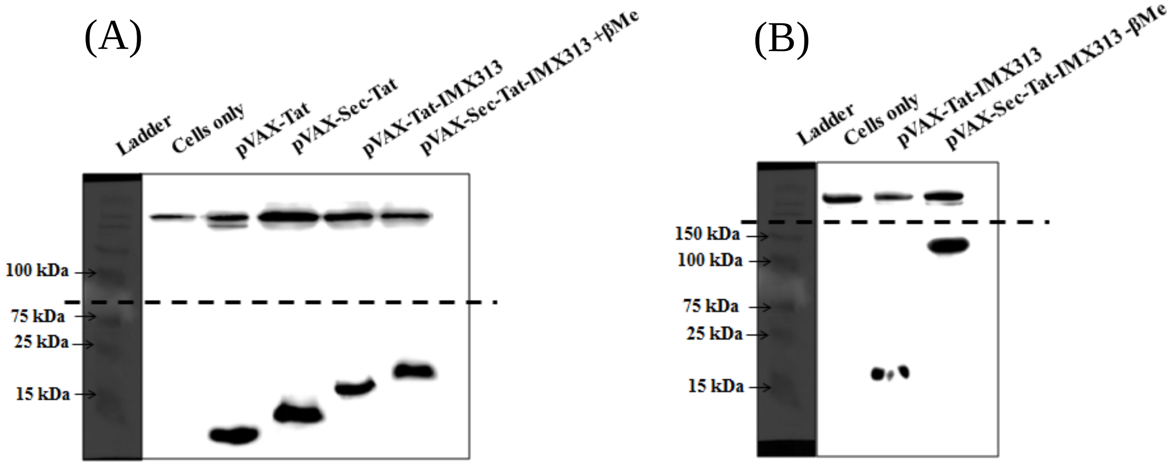

Supplementary Figure S1.Tat expression. (A) reducing Western blot analysis of Tat in cell lysates from HEK293T cells transfected with plasmid DNA encoding the different forms of Tat (tracks 2-5) and (B) non-reducing Western blot analysis of Tat in supernatant fluids of HEK293T cells transfected with pVAX-Tat-IMX313 (track 2) or pVAX-sTat-IMX313 (track 3) DNA. The dotted lines indicate the positions where the blots were cropped for figure IB and IC in the main manuscript.

(A)

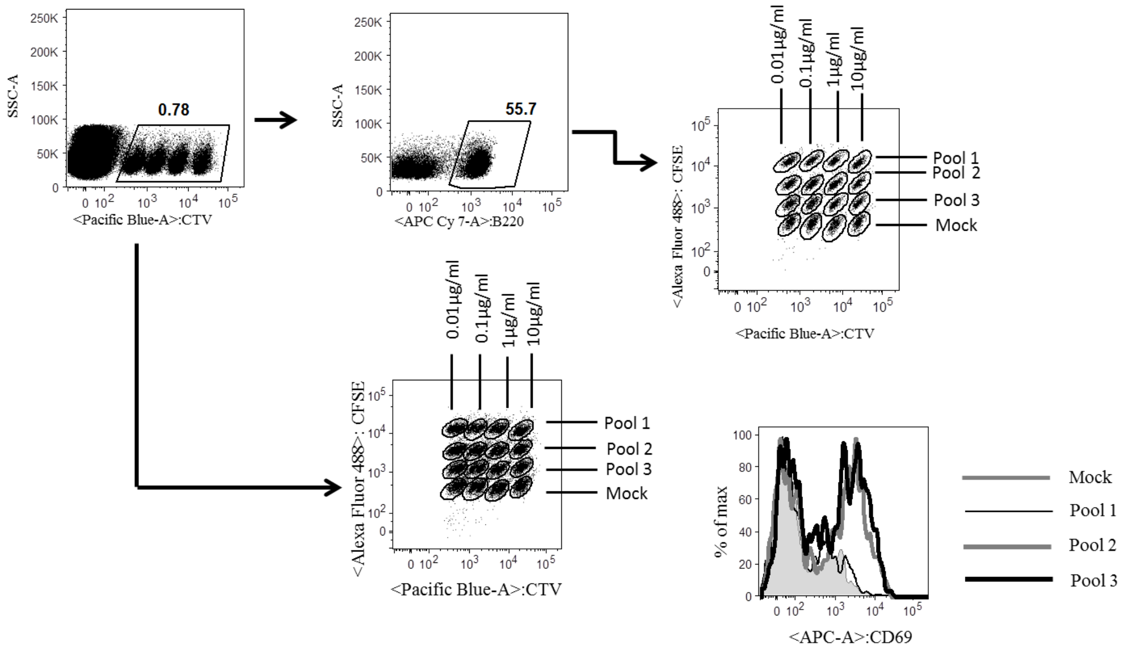

(B)

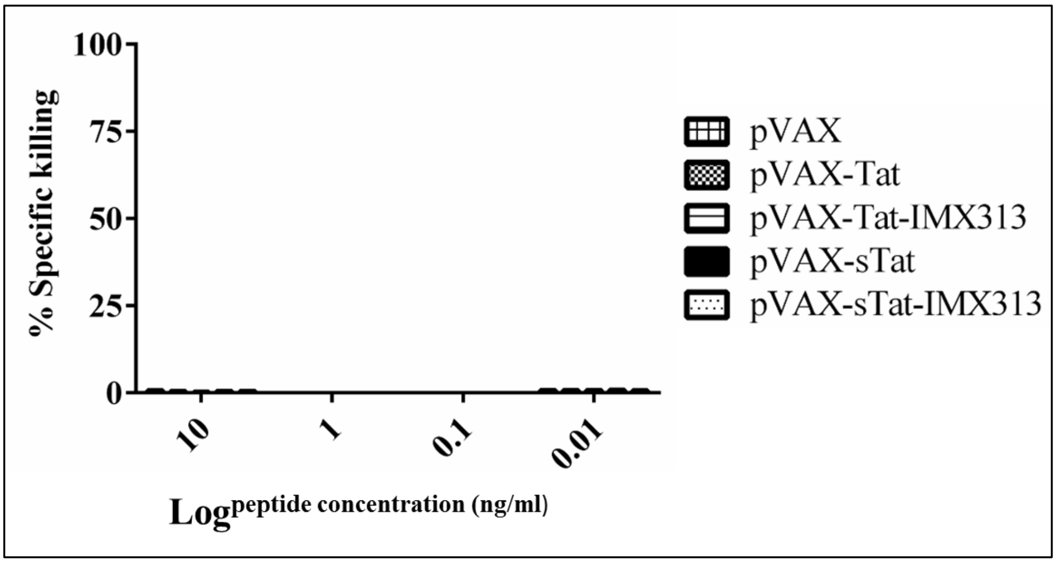

Supplementary Figure S2. Gating strategy for the FTA analysis conducted in Fig 2B and 2C. RBC-depleted splenocytes from naïve mice were labelled with titrated concentrations of CTV or CFSE dyes, and pulsed for 4h at 37 °C in 5% CO_2 with 3 Tat peptide pools (pool 1-3) at concentrations ranging from 10µg/ml to 0.01µg/ml or media only

(mock control) before adoptive transfer into vaccinated mice. Representative plots depict the gating strategy for FTA analysis on doublet discriminated lymphocytes from a Tat DNA vaccinated animal (A). Total FTA targets were delineated based on CTV labelling and the total percentage of target cells in each of the peptide pulsed-clusters relative to the mock clusters was analysed to determine the magnitude of *in vivo* killing of FTA cells (B). To determine the magnitude of Th cell responses, B220^{+} cells within the FTA were gated and CD69 up-regulation was determined on peptide pulsed B cells relative to mock B cells within the FTA.

Supplementary materials

Primers used to amplify Tat inserts and quantify EcoHIV RNA levels

a) For pVAX-Tat

1- Tat-forward: GTGCTAGCGCCAGCATGGAACCCGTGGACCCCAGAC

2- Tat- reverse: ACGAATTCGTCCTCGGGGTCTGTCTCTGTC

b) For pVAX-sTat

3- secr tat CMV fwd, forward: GAGAGAAAGCTT ATGGAACCCGTGGACCCC

4- secr tat CMV rev, reverse: GAGAGAGAATTCTCAGTCCTCGGGGTCTGT

c) For pVAX-sTat-IMX313

5- secr tat CMV fwd: GAGAGAAAGCTTATGGAACCCGTGGACCCC

6-Wt_Tat_nostop reverse:GAGAGAGAATTCGTCCTCGGGGTCTGTCTCTGTC

d) For pVAX-Tat-IMX313

7- Wt_Tat_no stop forward:TCT CTC GCT AGC GCC ACC ATG GAA CCC GTG GAC CCC AGA C

8- New Tat_IMX313 con_Rev-2: GAGAGACTCGAGTCACTCTTTGCTCAGGCCCTGC

e) For EcoHIV qPCR

9-RPL13a FWD_forward: GAGGTCGGGTGGAAGTACCA

10-RPL13a REV_reverse: TGCATCTTGGCCTTTTCCTT

11-MLV FWD_forward: TAGGGCCAAACCCCGTTCTG

12-MLV REV_reverse: GCCGGTGGAAGTTGGGTAGG
